# Potential Added Value of ^18^F-FDG PET Metabolic Parameters in Predicting Disease Relapse in Type 1 Autoimmune Pancreatitis

**DOI:** 10.1186/s12876-023-03113-7

**Published:** 2024-01-17

**Authors:** Shengxin Chen, Guanyun Wang, Lang Wu, Dexin Chen, Kaixuan Fang, Wenjing Liu, Baixuan Xu, Ya-qi Zhai, Mingyang Li

**Affiliations:** 1 Department of Gastroenterology and Hepatology, The First Medical Center, Chinese PLA General Hospital, 28 Fuxing Road, Haidian District, Beijing, 100853 China; 2 Graduate School, Chinese PLA General Hospital, 28 Fuxing Road, Haidian District, Beijing, 100853 China; 3 Department of Nuclear Medicine, The First Medical Center, Chinese PLA General Hospital, 28 Fuxing Road, Haidian District, Beijing, 100853 China; 4 Nuclear Medicine Department, Beijing Friendship Hospital, Capital Medical University, 95 Yong’an Road, Xicheng District, Beijing, 100050 China

**Keywords:** ^18^F-fluorodeoxyglucose positron emission tomography, Logistic model, metabolic parameters, Relapse, Type 1 autoimmune pancreatitis

## Abstract

**Background:**

The predictive value of ^18^F-fluorodeoxyglucose positron emission tomography/computed tomography (^18^F-FDG PET/CT) metabolic parameters for predicting AIP relapse is currently unknown. This study firstly explored the value of ^18^F-FDG PET/CT parameters as predictors of type 1 AIP relapse.

**Methods:**

This multicenter retrospective cohort study analyzed 51 patients who received ^18^F-FDG PET/CT prior to treatment and did not receive maintenance therapy after remission. The study collected baseline characteristics and clinical data and conducted qualitative and semi-quantitative analysis of pancreatic lesions and extrapancreatic organs. The study used three thresholds to select the boundaries of pancreatic lesions to evaluate metabolic parameters, including the maximum standard uptake value (SUV_max_), mean standard uptake value (SUV_mean_), total lesion glycolysis (TLG), metabolic tumor volume (MTV), and tumor-to-normal liver standard uptake value ratio (SUVR). Univariate and multivariate analyses were performed to identify independent predictors and build a recurrence prediction model. The model was internally validated using the bootstrap method and a nomogram was created for clinical application.

**Results:**

In the univariable analysis, the relapsed group showed higher levels of SUV_max_ (6.0 ± 1.6 vs. 5.2 ± 1.1; *P* = 0.047), SUVR (2.3 [2.0–3.0] vs. 2.0 [1.6–2.4]; *P* = 0.026), and TLG_2.5_ (234.5 ± 149.1 vs. 139.6 ± 102.5; *P* = 0.020) among the ^18^F-FDG PET metabolic parameters compared to the non-relapsed group. In the multivariable analysis, serum IgG_4_ (OR, 1.001; 95% CI, 1.000–1.002; *P* = 0.014) and TLG_2.5_ (OR, 1.007; 95% CI, 1.002–1.013; *P* = 0.012) were independent predictors associated with relapse of type 1 AIP. A receiver-operating characteristic curve of the predictive model with these two predictors demonstrated an area under the curve of 0.806.

**Conclusion:**

^18^F-FDG PET/CT metabolic parameters, particularly TLG_2.5_, are potential predictors for relapse in patients with type 1 AIP. A multiparameter model that includes IgG4 and TLG2.5 can enhance the ability to predict AIP relapse.

## Introduction

Autoimmune pancreatitis (AIP) is a rare form of chronic pancreatitis that was first proposed by Yoshida et al. [1] in 1995. The clinical profile of AIP includes enlargement of the pancreas, irregular narrowing of the pancreatic duct, and the involvement of multiple organs [2]. Type 1 AIP is an immunoglobulin (Ig) G4-related disease with increased serum IgG_4_ levels and a good response to corticosteroids. It is a benign relapsing disease with a treatment rate of 97.9%; however, its overall relapse rate ranges from 20 to 60% [3–6]. Furthermore, relapse seriously affects the lives of patients and comprises a heavy psychological burden; therefore, it is necessary to identify predictors of relapse during clinical diagnosis and treatment [6].

Unfortunately, there is no definite consensus regarding the predictive factors for AIP relapse. Some studies have reported that initially high serum IgG_4_ levels, persistently high serum IgG_4_ levels after treatment, diffuse enlargement of the pancreas, proximal IgG_4_ sclerosing cholangitis, and extensive multiorgan involvement may be risk factors of relapse [5, 7–10]. Recently, ^18^F-fluorodeoxyglucose positron emission tomography/computed tomography (^18^F-FDG PET/CT) metabolic parameters have been found to have good clinical value for determining the prognosis of many diseases [11, 12]. ^18^F-FDG PET/CT is advantageous when used for determining the differential diagnosis, performing early evaluation of the involved organ location, evaluating the lesion severity, and monitoring the response to AIP treatment [13]. However, the predictive value of ^18^F-FDG PET/CT for AIP relapse is unknown. This study is the first to explore the predictive value of ^18^F-FDG PET/CT metabolic parameters to identify patients at risk for relapse before treatment.

## Methods

### Study subjects

Consecutive patients with type 1 AIP who underwent an ^18^F-FDG PET/CT examination before treatment and an interventional examination were included in the study from two medical center, PLA General Hospital First Medical Center and PLA General Hospital Fifth Medical Center. The inclusion criteria were as follows: type 1 AIP conforming to the International Consensus Diagnostic Criteria for autoimmune pancreatitis (ICDC) [14]; and underwent an ^18^F-FDG PET/CT examination before treatment. The exclusion criteria were as follows: complicated diseases that interfere with ^18^FDG PET/CT imaging, such as malignancies and systemic autoimmune diseases; receiving maintenance therapy; pancreatectomy after PET/CT examination, and loss to follow-up. Medical data including basic demographics, history, laboratory data before treatment, and initial treatment (initial induction phase after PET/CT) of each patient were retrospectively collected from May 2009 to June 2019 from the electronic medical record database.

The study was approved by the Institutional Review Board of the Institutional Ethics Committee of the General Hospital of the People’s Liberation Army (No. S2022–428-01) with the requirement for obtaining informed consent waived.

### Follow-up

Patients were continuously followed-up using the outpatient service system and telephone interviews. All patients were followed-up for at least 3 years unless relapse was diagnosed. Relapse was defined as the development of imaging findings or liver test abnormalities consistent with a new or worsening inflammatory process [6, 15]. Symptoms without biochemical or radiographic abnormalities and isolated serum IgG_4_ elevation alone were not sufficient to diagnose relapse. However, for patients with previously documented biliary tract involvement, relapse was diagnosed without imaging findings when symptoms presented as obstructive jaundice and liver test results were more than three-times the upper limit of normal (ULN) [6, 16]. Relapse involving other organs was diagnosed based on pathology results or the typical imaging appearance [6, 17]. Maintenance therapy was defined as the regular use of low-dose glucocorticoids for more than 3 years after symptom remission. After initial steroid induction phase, patients who receiving maintenance therapy irregularly, discontinued maintenance therapy, or did not receive any maintenance therapy were defined as not receiving maintenance therapy.

### Image acquisition

All patients underwent ^18^F-FDG PET/CT (Biograph 64, Siemens Healthineers, Marburg, Germany; Discovery VCT, GE Healthcare, Chicago, IL, USA). Radiopharmaceutical ^18^F-FDG was synthesized using a cyclotron (HM-20; Sumitomo, Tokyo, Japan) and an automatic chemical synthesizer (Beijing PET-FDG-IT-NC) at the PLA General Hospital. The radiochemical purity was more than 95%. PET/CT studies were conducted according to the European Association of Nuclear Medicine guidelines [18]. Patients were instructed to fast for more than 6 hours, rest for at least 30 minutes in a quiet waiting room, and maintain a blood glucose level less than 11.1 mmol/L before intravenous administration of ^18^F-FDG. PET scanning was performed at least 45 minutes after the intravenous injection of ^18^F-FDG (3.5–4.5 MBq/kg) from the skull to the upper thigh in the free-breathing mode. The parameters of low-dose CT were as follows: rotation, 0.8; current, 100 mA; voltage, 120 to 140 kV; layer thickness, 3 to 5 mm; and pitch, 1. The PET parameters were as follows: three-dimensional mode, 2 to 2.5 minutes per bed position (30% overlap); 4 to 5 bed positions per person; Gaussian filter half-height width, 4.0 mm; 3 iterations; and 21 subsets. The ordered subset expectation maximization algorithm and CT attenuation correction were used to reconstruct the images.

### Image analysis

^18^F-FDG PET/CT images were analyzed intensively using the Advantage Workstation 4.6 (GE Healthcare). All images were independently read by two well-trained nuclear medicine physicians who were unaware of the clinical information of the patients. Any opinion differences were resolved through discussion. Images were evaluated using qualitative and semiquantitative methods. Lesions were defined as areas with abnormal uptake of ^18^F-FDG during PET imaging and/or abnormal density during CT imaging. During the qualitative analysis, the distribution of ^18^F-FDG in pancreatic lesions was classified as diffuse/multifocal or segmental/focal. Focal lesions were defined as lesions limited to one region of the head, body, or tail. Segmental lesions were defined as those in two consecutive regions. Multifocal lesions were defined as those in more than one noncontiguous region. The pattern of ^18^F-FDG activity in the region of interest of the pancreas was classified as homogeneous or heterogeneous. A heterogeneous pattern was defined as the accumulation of visual differences in ^18^F-FDG within the region of interest or between the inner and marginal regions [19]. Extrapancreatic organs were defined as areas with abnormal ^18^F-FDG uptake and excluded the physiologically increased ^18^F-FDG uptake regions of the myocardium, renal pelvis, intestine, and bladder. All organs outside the pancreas and involved lymph node regions were counted. During the semiquantitative analysis, we used three thresholds to select the boundaries of pancreatic lesions to achieve better predictive ability (SUV_max_ threshold, 40, 42%; SUV fixed threshold, ≥2.5). We placed a three-dimensional volume of interest over the pancreatic lesions while carefully avoiding the inclusion of physiological uptake. Using this volume of interest, the maximum standardized uptake value (SUV_max_), mean standard uptake value (SUV_mean_), metabolic tumor volume (MTV), and total lesion glycolysis (TLG) (TLG = SUV_mean_ × MTV) were calculated. A region of interest with a diameter of 3 cm was automatically fixed in the right lobe of the liver away from the hepatic hilum area. Then, the SUV_mean_ of the normal liver and pancreas-to-normal liver standard uptake value ratio (SUVR) (defined as SUV_max_ of the pancreas/SUV_mean_ of the normal liver parenchyma) were measured.

### Statistical analysis

The statistical analysis was performed using Statistical Product and Service Solutions 26.0 (SPSS Inc., Chicago, IL, USA) and R software (version 4.0.2; Bell Laboratories, Murray Hill, NJ, USA). Continuous data are described as the mean ± standard deviation or median with interquartile range. Categorical data are described as frequencies with proportions. The association between potential predictors and relapse was first evaluated by univariate analyses, including the Student t-test and nonparametric Mann-Whitney test for continuous data and the chi-square and Fisher exact tests for categorical data. Potential predictors associated with the relapse of type 1 AIP in univariable analysis (*P* < 0.1) were included in the multivariable logistic regression analysis, then the backward stepwise elimination method was used to identify independent predictors. We opted for a cutoff *P* of 0.10 because of the small sample size. Multivariate logistic regression analysis was used to construct a predictive model. To assess the prediction model’s discrimination and calibration, we conducted a receiver-operating characteristic curve (ROC) analysis and Hosmer-Lemeshow goodness-of-fit test. We also calculated accuracy, sensitivity, specificity, and cutoff value. Internal validation was performed using the bootstrap method. Based on the multivariable logistic regression analysis, we developed a nomogram to facilitate clinical use of the model. All statistical tests were two-tailed, and *P* < 0.05 was considered statistically significant.

## Results

### Baseline characteristics and clinical variables

**Fig. 1:**
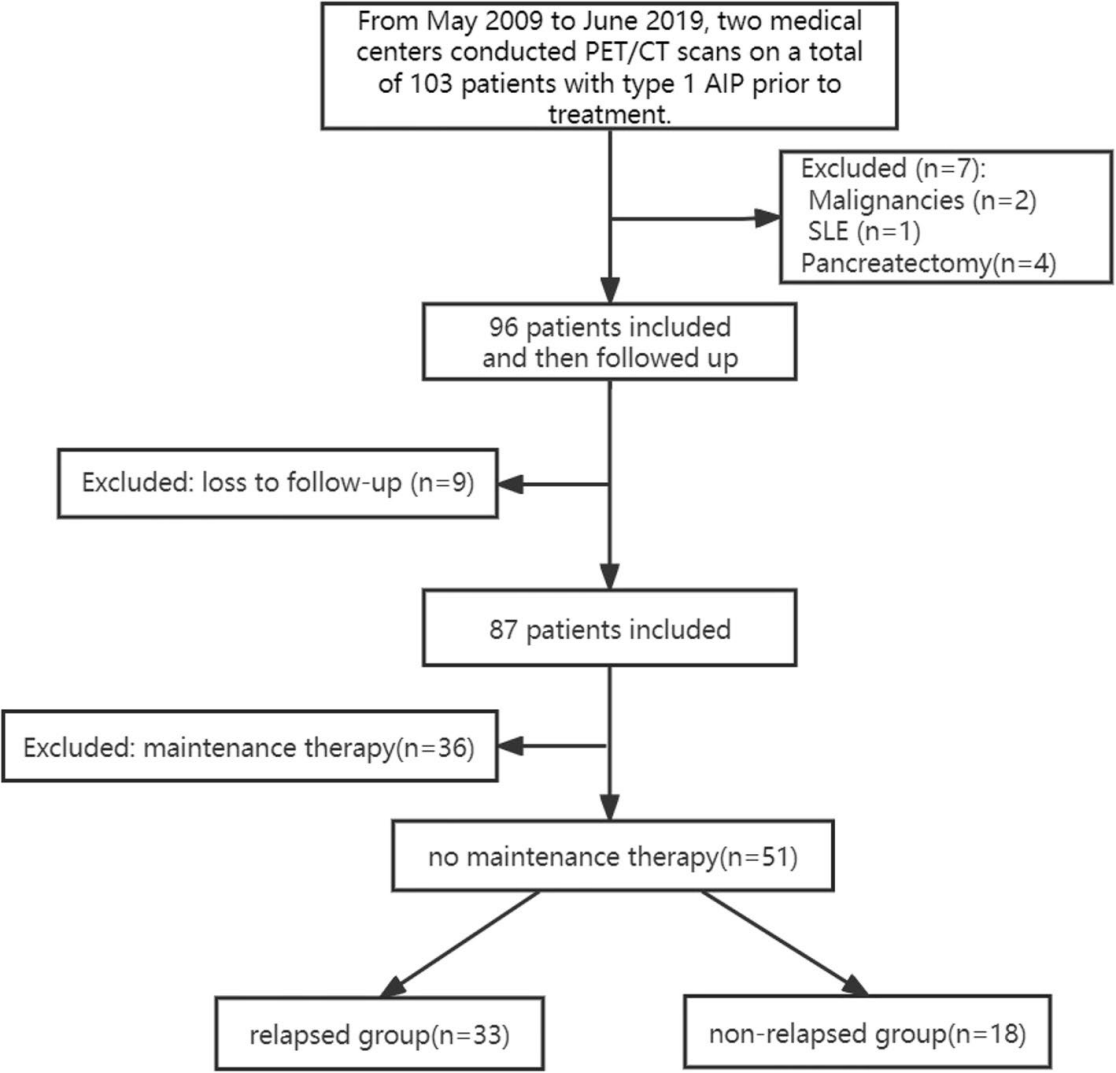
Flowchart of the inclusion and exclusion process of study subjects. AIP, autoimmune pancreatitis; SLE, systemic lupus erythematosus. Malignancies include diffuse large B-cell lymphoma and metastatic gastric carcinoma

Of 103 consecutive patients with type 1 AIP who underwent ^18^F-FDG PET/CT before treatment and an interventional examination, 52 were excluded because of malignancies (diffuse large B-cell lymphoma and metastatic gastric carcinoma; *n* = 2), systemic lupus erythematosus (*n* = 1), treatment with pancreatectomy (*n* = 4), loss to follow-up (*n* = 9), and glucocorticoids maintenance therapy (*n* = 36). The final cohort comprised 51 patients (37 males and 14 females) with a mean age of 62.7 years (±11.8 years); 33 of these patients experienced relapse and 18 did not (Fig. 1). The average time from PET to the initiation of steroid therapy was 7.4 days (range 3–19 days). The overall relapse rate of this cohort was 64.7%. The median recurrence time was 26 months (range 4–83 months), and the relapse manifestations included recurrent jaundice (*n* = 18), pancreatic imaging abnormalities (pancreatic enlargement or pancreatic duct stenosis; *n* = 8), extrapancreatic organ involvement (parotid enlargement, submandibular/lacrimal gland enlargement, IgG_4_-related hypophysitis, retroperitoneal fibrosis, lymph node enlargement; *n* = 5), and recurrent acute pancreatitis (*n* = 2). The median follow-up time of the non-relapsed group was 57 months (range 36–106 months). In the initial induction phase, 35 out of 51 patients (68.6%) were prescribed steroids (Prednisone/Methylprednisolone), with a median dose of 40 mg/day (range 25–67.5 mg/day), and the average duration of medication was 3.5 weeks (range 2–8 weeks). The detailed clinical characteristics of the two groups were shown in Table 1. The serum IgG_4_ values (1190.0 [556.5–2420.0] vs. 588.0 [319.8–964.8]; *P* = 0.008) of the relapsed group were significantly higher than that of the non-relapsed group. There were no differences in age, sex, body mass Index (BMI), history, clinical symptoms, and some laboratory test results (levels of white blood cells, creatinine, total bilirubin, and lactate dehydrogenase) between the relapsed and non-relapsed groups.

**Table 1:** Comparison of clinical characteristics between the relapsed group and the non-relapsed group

	Relapsed group (*N* = 33)	Non-relapsed group (*N* = 18)	*P* value
Age, years	62.7 ± 13.8	62.6 ± 7.4	0.985
Sex			
Male, n (%)	25(75.8)	12(66.7)	0.714
Female, n (%)	8(24.2)	6(33.3)	
BMI	22.2 ± 2.5	22.3 ± 2.1	0.831
**Past history**			
Diabetes, n (%)	7(21.2)	3(16.7)	0.983
Smoke, n (%)	16(48.5)	9(50)	0.918
Alcohol, n (%)	15(45.5%)	11(61.1)	0.285
**Clinical symptoms**			
Obstructive jaundice, n (%)	23(69.7)	11(61.1)	0.534
Abdominal pain, n (%)	19(57.6)	8(44.4)	0.369
Acute pancreatitis, n (%)	1(3.0)	2(11.1)	0.583
Lose weight, n (%)	15(45.5)	8(44.4)	0.945
**Laboratory tests**			
IgG4, mg/dL	1190.0(556.5–2420.0)	588.0(319.8–964.8)	**0.008**
TB, umol/L	71.2(30.8–166.3)	69.6(17.6–194.0)	0.921
Cr, umol/L	63.3(54.7–73.5)	64.7(51.4–67.9)	0.775
WBC, × 109/L	6.1(5.3–7.7)	5.2(4.6–6.7)	0.200
LDH, U/L	155.9(128.3–187.0)	153.9(137.2–186.5)	0.937
**Initial treatment**			
Glucocorticoids alone, n (%)	13(41.9)	2(11.1)	–
Glucocorticoids+Immunosuppressant, n (%)	7(22.6)	8(44.4)	–
Glucocorticoids+Biliary drainage, n (%)	5(16.1)	0(0)	–
Biliary drainage, n (%)	3(9.7)	1(5.6)	–
Biliary drainage+ Immunosuppressant, n (%)	3(9.7)	4(22.2)	–
Immunosuppressant, n (%)	2 (6.5)	3 (16.7)	–

* Bolded values indicate that the difference has statistical significance; Lose weight: weight reduction of more than 5 kg within 3 months due to AIP; *TB* Total bilirubin, *Cr* Creatinine, *WBC* White blood cell, *LDH* Lactate dehydrogenase

### Comparison of ^18^F-FDG PET metabolic parameters and qualitative variables of the relapsed group and the non-relapsed group

The comparison between the relapsed and non-relapsed groups for ^18^F-FDG PET metabolic parameters and qualitative variables is summarized in Table 2. Regarding semiquantitative analysis, there were significant differences in the SUV_max_ (6.0 ± 1.6 vs. 5.2 ± 1.1; *P* = 0.047) and SUVR (2.3 [2.0–3.0] vs. 2.0 [1.6–2.4]; *P* = 0.026) between the relapsed and non-relapsed groups. When the threshold method was based on SUV ≥2.5, TLG_2.5_ (234.5 ± 149.1 vs. 139.6 ± 102.5; *P* = 0.020) in the relapsed group was higher than that in the non-relapsed group; there was no significant difference in SUV_mean2.5_ and MTV_2.5_ between the two groups. When the threshold method was based on 40 and 42% of the SUV_max_, there were no differences in SUV_mean_, TLG, and MTV between the relapsed and non-relapsed groups. According to qualitative analysis, the proportion of diffuse/multifocal distribution of FDG activity (24 [72.7%] vs. 7 [38.9%], *P* = 0.018) in the relapsed group was higher than those in the non-relapsed group. There was no significant difference in pattern of FDG activity, total number of extrapancreatic organs, extrapancreatic organ types, and involved lymph node regions between the two groups.

**Table 2:** Comparison of ^18^F-FDG metabolic parameters and qualitative variables between the relapsed group and the non-relapsed group

	Relapsed group	Non-relapsed group	*P* value
	(*N* = 33)	(*N* = 18)	
**Semiquantitative analysis**			
**Pancreatic lesions**			
SUV_max_, g/ml	6.0 ± 1.6	5.2 ± 1.1	**0.047**
SUVR	2.3(2.0–3.0)	2.0(1.6–2.3)	**0.026**
SUV_mean2.5_, g/ml	3.4(3.1–3.7)	3.2(2.9–3.6)	0.515
SUV_mean40%_, g/ml	3.3 ± 0.9	2.9 ± 0.7	0.108
SUV_mean42%_, g/ml	3.5 ± 0.9	3.0 ± 0.7	0.069
MTV_2.5_, cm^3^	70.2(33.9–100.2)	24.9(18.6–72.4)	0.064
MTV_40%_, cm^3^	80.4(44.7–98.3)	65.8(31.6–136.1)	0.608
MTV_42%_, cm^3^	74.2(41.0–90.0)	54.8(28.0–126.9)	0.503
TLG_2.5_, g/ml×cm^3^	234.5 ± 149.1	139.6 ± 102.5	**0.020**
TLG_40%_, g/ml×cm^3^	256.9(153.9–343.7)	164.9(90.7–351.2)	0.419
TLG_42%_, g/ml×cm^3^	236.7(141.3–320.4)	154.1(80.9–330.2)	0.315
**Qualitative analysis**			
**Pancreatic lesions**			
Pattern of FDG activity, n (%)			0.782
Homogeneous	17(51.5)	10(55.6)	
Heterogeneous	16(48.5)	8(44.4)	
Distribution of FDG activity, n (%)			**0.018**
Diffuse/Multifocal	24(72.7)	7(38.9)	
Segmental/ Focal	9(27.3)	11(61.1)	
**Extrapancreatic organ**			
Lung, n (%)	12(36.4)	7(38.9)	0.397
Salivary gland, n (%)	10(30.3)	2(11.1)	0.231
Biliary duct, n (%)	22(51.2)	18(40.9)	0.212
Kidney, n (%)	4(12.1)	1(5.6)	0.794
Total number	2.9 ± 1.8	2.6 ± 1.5	0.637
**Region of lymph nodes**			
Cervical, n (%)	9(27.2)	4(22.2)	0.953
Thoracic, n (%)	17(51.5)	9(50.0)	0.918
Abdominal, n (%)	15(45.5)	11(61.1)	0.285
Pelvic, n (%)	4(12.1)	1(5.6)	0.794

* Text in bold indicates a statistically significant difference; SUV_max_: Max standard uptake value; SUV_mean_: Mean standard uptake value; *MTV* Metabolic tumor volume, *TLG* Total lesion glycolysis, *SUVR* standard uptake value ratio

### Multivariate analysis and constructing predictive model

**Fig. 2:**
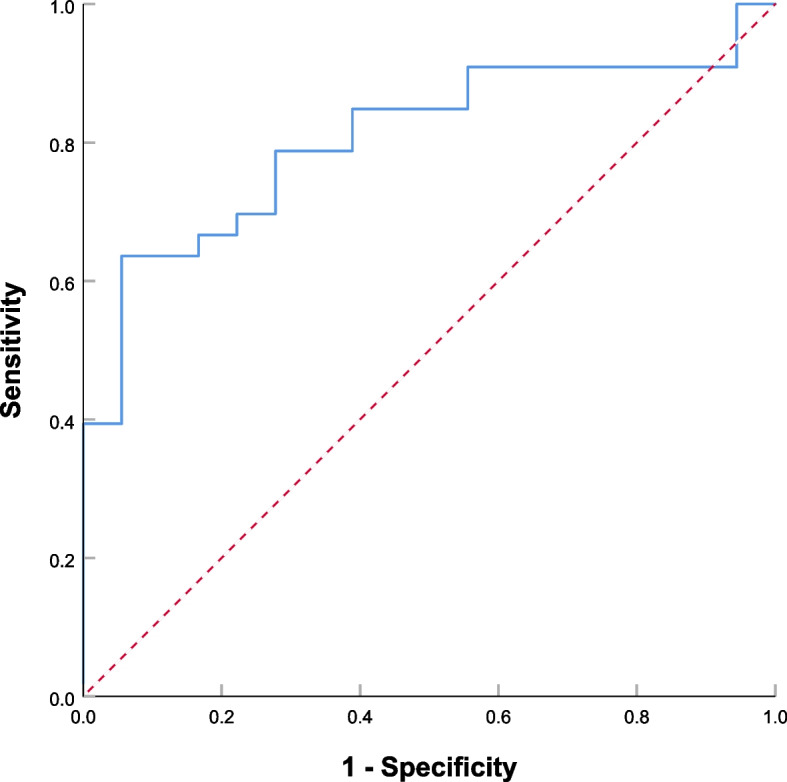
Receiver-operating characteristic curve of the multivariable regression model for predicting relapse of type 1. Area under the curve, 0.806

**Fig. 3:**
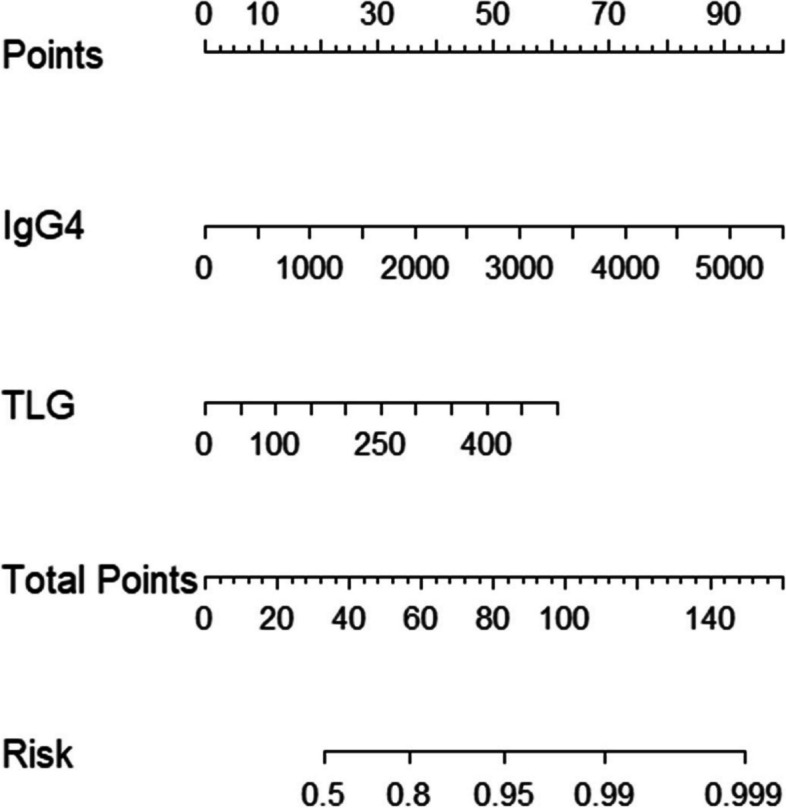
Nomogram to predict the probability of AIP recurrence

**Fig. 4:**
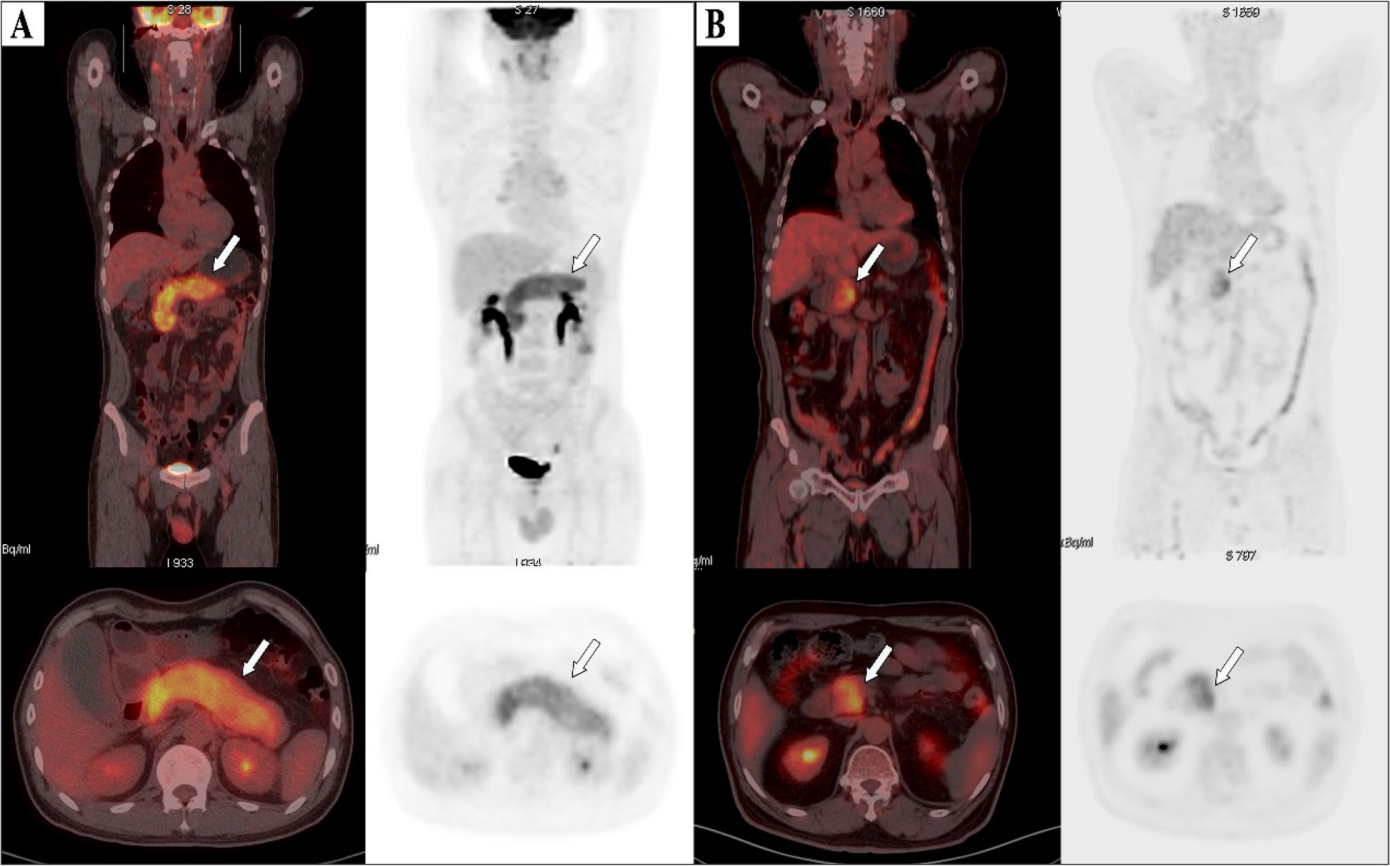
^18^F-FDG PET-CT images of patients. **A** Image of a 61-year-old male patient with type 1 AIP, demonstrating diffuse enlargement of the pancreas. (arrows). The serum IgG4 level was 2260.0 mg/dL, and the TLG_2.5_ was 413.8 g/ml×cm^3^. According to the Nomogram, his relapse probability is more than 95%. The patient discontinued steroid use during the maintenance phase, and 7 months later, he relapsed, with imaging showing diffuse re-enlargement of the pancreas. **B** Image of a 60-year-old male patient with type 1 AIP, showing focal enlargement in the head of the pancreas (arrows). The serum IgG4 level was 980 mg/dL, and the TLG_2.5_ was 92.7.0 g/ml×cm^3^. According to the Nomogram, the patient’s relapse probability is less than 50%. After symptom relief, the patient did not receive maintenance treatment, and has been followed up for 4 years without relapse

In multivariable analysis, potential predictors with *P* < 0.1 by univariate analysis, such as serum IgG_4_, SUV_max_, SUVR, TLG_2.5_, MTV_2.5_, SUV_mean42%_, and distribution of FDG activity, were included in multivariable logistic regression analysis, and then used backward stepwise elimination method to exclude variables with *P* > 0.05 from the model (Table 3). Eventually, the following 2 variables remained significantly associated with relapse (*P* < 0.05): serum IgG_4_ (OR, 1.001; 95% CI, 1.000–1.002; *P* = 0.014) and TLG_2.5_ (OR, 1.007; 95% CI, 1.002–1.013; *P* = 0.012) (Table 4). We constructed a predictive model based on serum IgG_4_ and TLG_2.5_; the ROC curve showed an area under the curve (AUC) of 0.806 (Fig. 2), and accuracy of 70.6%, sensitivity of 0.788, and specificity of 0.722 (Table 5). The Hosmer-Lemeshow goodness of fit test showed reasonable calibration of the predictive model (*P* = 0.131). To validate the model, we used the bootstrap method with 1000 iterations. The bootstrap analysis showed that the model had good internal validity, with a mean AUC of 0.81 (95% CI: 0.74–0.87). Subsequently, a nomogram was created based on the two independent factors linked with type 1 AIP relapse (as shown in Fig. 3). The algorithm assigned points to each factor separately, and an increase in total points corresponded to an increased probability of relapse. Two typical cases and PET/CT images were shown in Fig. 4.

**Table 3:** Univariable (*P* < 0.1) and multivariable analyses of the association with relapse-free survival in patients with type 1 AIP

	Univariable analysis		Multivariable analysis	
	Odds ratio (95% CI)	*P* value	Adjusted Odds ratio (95% CI)	*P* value
Clinical variables				
IgG_4_, mg/dL	1.001(1.000–1.002)	0.008	1.007 (1.000–1.002)	0.014
PET semiquantitative parameter				
SUV_max_, g/ml	1.568(0.991–2.483)	0.047		
SUVR	2.683(1.009–7.137)	0.026		
SUV_mean42%_, g/ml	1.990(0.931–4.257)	0.069		
MTV_2.5_, cm^3^	1.018(1.001–1.035)	0.064		
TLG_2.5_, g/ml×cm^3^	1.006(1.001–1.010)	0.020	1.001 (1.002–1.013)	0.012
PET qualitative variables				
Distribution of FDG activity				
Diffuse/Multifocal	4.190(1.239–14.174)	0.018		
Segmental/Focal	Reference category			

* SUV_max_: Max standard uptake value; SUV_mean_: Mean standard uptake value; *MTV* Metabolic tumor volume, *TLG* Total lesion glycolysis, *SUVR* standard uptake value ratio

**Table 4:** Multivariable regression analysis of predictors for relapse in patients with type 1 AIP

	*β*	*Wald*	Odds ratio	95% Confidence interval	*P* value
**TLG**_**2.5**_	0.007	6.323	1.007	1.002–1.013	**0.012**
**IgG**_**4**_	0.001	5.996	1.001	1.000–1.002	**0.014**

*TLG* Total lesion glycolysis

**Table 5:** Assessment of the predictive efficiency of relapse using ^18^F-FDG PET parameters, serological examinations, and a predictive model

Predictor	Cut-off value	Accuracy	AUC	Sensitivity	Specificity	Yoden Index
TLG_2.5_	213.9 (g/ml×cm^3^)	0.608	0.678	0.606	0.788	0.384
IgG4	946.5 (mg/dL)	0.647	0.726	0.667	0.778	0.445
Model		0.706	0.806	0.788	0.722	

*TLG* Total lesion glycolysis

## Discussion

To the best of our knowledge, this is the first study to explore ^18^F-FDG PET/CT metabolic parameters to predict type 1 AIP relapse. We found that pretreatment ^18^F-FDG PET/CT metabolic parameters may be useful predictors of relapse, and that TLG had better ability to predict type 1 AIP relapse when the SUV fixed threshold was 2.5. We also established a multiparameter model based on IgG_4_ and TLG_2.5_ with an estimated predictive performance of AUC 0.806 that could significantly improve the ability to predict type 1 AIP relapse.

Type 1 AIP, also known as lymphoplasmacytic sclerosing pancreatitis, is the manifestation of pancreatic involvement in IgG_4_-related diseases [14, 20]. It is characterized by a high relapse rate ranging from 20 to 60% [3–6]. Our cohort showed that the relapse rates of patients who did not receive maintenance therapy with low doses of glucocorticoids were as high as 64.7%. AIP relapse increases the risk of pancreatic atrophy, resulting in significantly decreased pancreatic function [3, 5]. Although some risk factors for relapse have been proposed [5, 7–10], the predictors of relapse remain poorly understood [10].

The serum IgG_4_ level is an important serological marker in clinical practice. Several studies have found that increased serum IgG_4_ levels at diagnosis may predict relapse [9, 21, 22]. However, the relationship between IgG_4_ levels and relapse has been debated from different perspectives, and some studies have reported that these are not linked [6]. Our data demonstrate that serum IgG_4_ levels in the relapsed group were significantly higher than that in the non-relapsed group, and the optimal cutoff value was 946.5 mg/dL (approximately five times the ULN; OR, 1.001; 95% CI, 1.000–1.002). This is similar to the recommendation of the international consensus for the treatment of AIP, which suggests that a markedly increased serum IgG_4_ level (such as > 4-times the ULN) before treatment is a predictor of relapse [10]. Our results show that serum IgG_4_ levels had relatively high predictive specificity (0.778); however, the predictive sensitivity (0.667) of serum IgG_4_ alone is relatively low.

^18^F-FDG PET/CT is a noninvasive diagnostic tool that can provide anatomical morphology and metabolic information of the whole body with a single scan [18]. The prognostic value of ^18^F-FDG PET/CT metabolic parameters has been demonstrated for many diseases [11, 12, 23]. However, there are no reports of the prognosis determined using PET/CT for patients with AIP. We sought to explore whether ^18^F-FDG PET/CT could predict the recurrence of type 1 AIP. We found that, without low-dose glucocorticoid maintenance therapy, the ^18^F-FDG PET/CT metabolic parameters SUV_max_, SUVR, and TLG_2.5_ of the relapsed group were higher than those of the non-relapsed group in univariable analysis, suggesting that they may be potential risk factors for recurrence. Furthermore, based on multivariable analysis, we found that TLG_2.5_ (OR, 1.001; 95% CI, 1.002–1.013; AUC = 0.678) may be a better predictor of relapse among all ^18^F-FDG metabolic parameters. Type 1 AIP is an immune-mediated inflammatory disease involving lymphoplasmacytes that are rich in IgG_4_ infiltrates and fibrosis of the tissue [20]. Because of the increased ^18^F-FDG accumulation in inflammatory lesions, ^18^F-FDG PET/CT may contribute to the presence of pancreatic and extrapancreatic organs in AIP [24, 25]. TLG is a semi-quantitative parameter that combines metabolism and volume and can reflect the activity of glucose metabolism in the overall lesion [26]. Our results showed that patients with higher TLG are more likely to experience recurrence. TLG, as a volumetric parameter of ^18^F-FDG PET, may reflect a wider accumulation of IgG_4_ in the pancreas, greater glucose consumption in the lesions, and stronger activity. Therefore, these patients may require longer treatment and more effective medications to prevent recurrence. TLG_2.5_ had better predictive specificity (0.778); however, the predictive sensitivity of TLG_2.5_ (0.606) was relatively poor according to our results. To enhance the predictive performance, we identified two independent predictors, IgG_4_ and TLG_2.5_, that were associated with relapse, and developed a multiparameter model incorporating both factors. The use of multiple parameters in the model served to overcome the limitations of relying on a single parameter alone. The Hosmer-Lemeshow test and internal validation results indicate that the model has reasonable calibration. To facilitate clinical application, we have created a nomogram that predicts the likelihood of AIP recurrence based on TLG_2.5_ and IgG_4_ values.

We analyzed pancreatic and extrapancreatic organs by qualitative method to show other findings. Our data showed that the proportion of diffuse/multifocal pancreatic uptake was significantly higher in the relapsed group than in the non-relapsed group. Both qualitative and semi-quantitative (TLG_2.5_) results also suggested that the larger the volume of pancreatic involvement, the more extensive the inflammatory activity, and that the higher metabolic activity of the lesion may lead to a higher risk of AIP recurrence. This result is similar to the previous treatment consensus [10]. Although it was similar between groups, a nonstatistical difference in proportion of salivary gland involvement was higher in the relapsed group when compared with the non-relapsed group (10 [30.3%] vs. 2 [11.1%], *P* = 0.231). These results were similar to those of studies by Yasutaka and Kuruma et al., who found that type 1 AIP accompanied by salivary gland involvement predicted relapse [27, 28]. Previous studies have found that AIP with biliary tract involvement had a greater tendency to relapse; however, our results show that there was no significant difference in the proportion of biliary tract involvement between the two groups, which is contrary to our initial perception. The possible reasons for this discrepancy could be attributed to the poorer visualization of the bile ducts with ^18^F-FDG PET/CT as compared to MRI biliary imaging due to its lower sensitivity, along with possible factors such as sample bias and insufficient sample size. Even though our research suggests that the number of extrapancreatic organs with ^18^F-FDG uptake and affected lymph nodes before treatment were not distinctly different between the relapsed and non-relapsed groups, we must note that the distribution of systemic organs was sufficiently assessed using ^18^F-FDG PET/CT within our study population. These results are helpful in determining the degree of disease activity and differential diagnosis.

This study had some limitations. First, although this is the first study to explore ^18^F-FDG PET/CT metabolic parameters for predicting type 1 AIP relapse, the retrospective cohort study involving different treatment protocols may have a confounding effect on prognostication. In addition, the small sample size may limit the study results. Therefore, it is necessary to conduct large-scale, prospective validation studies among a homogeneous population of patients with AIP. Second, our study lacked external validation. Given the rarity of AIP and limited sample size, we were only able to perform internal validation, which nevertheless yielded promising results. We plan to gradually incorporate additional cases for external validation in the future. Third, although our study included patients from two medical centers and ensured similarity in the basic characteristics between treatment groups, it should be noted that the SUV is influenced by various factors, which may result in lack of repeatability of the model developed with metabolic parameters in clinical practice. In order to enhance the stability and repeatability of the model, we plan to incorporate additional PET image omics parameters, such as texture parameters, in our future studies.

In conclusion, a single PET/CT scan prior to treatment can aid in both diagnosing and assessing the distribution of lesions throughout the body, as well as provide a determination of the probability of recurrence. Among the ^18^F-FDG PET metabolic parameters, TLG_2.5_ is a better predictor of type 1 AIP relapse. Multiparameter models based on IgG4 and TLG_2.5_ can help to predict type 1 AIP relapse, and to some extent, screen patients who are more likely to benefit from maintenance therapy.

## Data Availability

The data sets used and analyzed during the current study are available from the corresponding author upon reasonable request.
